# Distribution and habitat of the Laotian Rock Rat *Laonastes aenigmamus* Jenkins, Kilpatrick, Robinson & Timmins, 2005 (Rodentia: Diatomyidae) in Vietnam

**DOI:** 10.3897/BDJ.2.e4188

**Published:** 2014-12-25

**Authors:** Dang Xuan Nguyen, Nghia Xuan Nguyen, Duy Dinh Nguyen, Tri Huy Dinh, Dinh Thuc Le, Duong Hai Dinh

**Affiliations:** † Institute of Ecology and Biological Resources, VAST, Hanoi, Vietnam; ‡ Phong Nha Ke Bang National Park, Quang Binh Province, Vietnam

**Keywords:** *Laonastes aenigmamus*, Laotian Rock Rat, Phong Nha - Ke Bang, Khammouane, limestone forests, Great Annamite.

## Abstract

The Laotian Rock Rat *Laonastes aenigmamus* Jenkins, Kilpatrick, Robinson & Timmins, 2005 was originally discovered in Lao People's Democratic Republic in 2005. This species has been recognized as the sole surviving member of the otherwise extinct rodent family Diatomyidae. *Laonastes aenigmamus* was initially reported only in limestone forests of Khammouane Province, Central Lao. A second population was recently discovered in Phong Nha Ke Bang National Park (PNKB NP), Quang Binh Province, Central Vietnam in 2011. The confirmed distribution range of *L. aenigmamus* in Vietnam is very small, approximately 150 km^2^, covering low karst mountains in five communes of Minh Hoa District, Quang Binh Province, at elevations between 250 and 400 m asl. The Laotian Rock Rat inhabits the lower part of steep karst towers with many rock boulders and crevices under tall limestone evergreen forest. They use small rock crevices for their dens. The natural habitat of this species in PNKB NP has been affected by selected timber harvesting, however, a complex 3-4 layer forest structure is retained. The Laotian Rock Rat is omnivorous, feeding on parts (leaves, buds, fruits and roots) of 18 plant species and also some insects (cicada, mantis, grasshopper). The population of this species in PNKB NP is seriously threatened with extinction due to its very restricted distribution, high hunting pressure, and habitat disturbance. *Laonastes aenigmamus* is listed in the IUCN Red List as endangered and in the Wildlife and Aquatic Red List of Lao, however, this species has not been listed in the Red Data Book or any conservation legislative documents of Vietnam.

## Introduction

The Laotian Rock Rat, *Laonastes aenigmamus*, was first discovered in 2005 in Lao People's Democratic Republic (Jenkins et al. 2005). The species was recognized as a surviving member of the otherwise extinct rodent family Diatomyidae, known from early Oligocene to late Miocene sites in Pakistan, India, Thailand, China, and Japan (Dawson et al. 2006). The Laotian Rock Rat was previously found only in limestone forests of Khammouane Province, Central Lao (Jenkins et al. 2005, Khotpathoom 2011, Vongsa 2010). In 2011, this species was recorded for the first time from Vietnam on the basis of four specimens trapped by local villagers in the buffer zone of Phong Nha Ke Bang National Park (PNKB NP), Central Vietnam (Nguyen et al. 2012). The discovery of a second population of Laotian Rock Rat in PNKB NP promotes opportunities for conservation for this enigmatic species. However, very little is known about the status and ecology of the Vietnamese population. During 2013 and 2014, we carried out 4 surveys in PNKB NP and adjacent areas to identify the distributional extent and habitat preference of the Laotian Rock Rat in Vietnam. In addition, threats to this species and its habitat were evaluated.

The PNKB NP is located in the western part of Quang Binh Province, which borders Khammouane Province of Lao. The national park is characterized by very specific topographic conditions consisting of precipitous karst ridges, which rise to elevations of around 400 m. Scattered among these ridges are narrow valleys and pockets of igneous rock formations. The limestone karst is almost entirely forested, apart from steep cliff faces. Forest clearance occurs only in flat valleys within the limestone massif and in lowland areas bordering it. The most widespread forest type in the PNKB NP is limestone evergreen forest, but there are also significant areas of lowland evergreen forest distributed on non-calcareous substrate in valleys among the limestone karsts (Le et al. 2012). The climate in PNKB NP area is tropical, hot and humid. The annual mean temperature ranges between 23^o^C and 25^o^C, with summer maximum of 41^o^C and winter minimum of 6^o^C. The high annual rainfall averages 2,000 - 2,500 mm, 88% falling between July and December, though there is rain in every month. The mean annual relative humidity is 84% (Le et al. 2012). Because of limestone topography, drainage is complex and there are few permanent water sources.

## Materials and methods

The range of the Laotian Rock Rat distribution was identified through village interviews and live-trapping. The village interview survey was conducted in three communes (Thuong Hoa, Hoa Son and Dan Hoa) of Minh Hoa District, Quang Binh Province. These communes were selected because only these communes contain primary or little affected limestone forest as preferable habitat of the Laotian Rock Rat and are situated close to locality where the first Vietnamese specimens of Laotian Rock Rat were collected in 2011. During the village interview survey, all previously trapped animals of Laotian Rock Rat or their remains were collected for further laboratory studies.

Based on the results of our village interview survey, 12 areas where local villagers have previously trapped the Laotian Rock Rat were selected for targeted field surveys using cage traps and box traps. After taking morphological characters, all live-trapped animals were released back into the wild at the place where they were trapped. Study of Laotian Rock Rat habitat was carried out in the same 12 areas using transect survey and plot survey techniques. Plots of 10 x 10 m were used for inventory of all trees with height more than 3m, plots of 4 x 4 m for inventory of bush trees of height from 0.5 m to 3 m, and plots of 1 x 1 m for inventory of herbs and tree seedlings less than 0.5 m high.

Food items of the Laotian Rock Rat were identified by examining the stomach contents of 10 preserved specimens, food remnants found in the species dens, and observation of live specimens in semi-wild conditions in natural habitat. Food identification was conducted by taxonomic experts based on morphological characters of food item remains. Threats to population and habitat of Laotian Rock Rat were evaluated based on interviews of local villagers and direct observation of threat signs (traps, hunters, logging, forest clearing, human encroachment) in the species distribution area.

## Taxon treatments

### Laonastes aenigmamus

Jenkins, Kilpatrick, Robinson & Timmins, 2005

#### Materials

- **Type status:** Other material. **Occurrence:** recordedBy: Nghia Xuan Nguyen; individualCount: 1; sex: male; **Location:** country: Vietnam; stateProvince: Quang Binh; verbatimLocality: Thuong Hoa Commune, Minh Hoa District; verbatimElevation: 200-350m; verbatimLatitude: 17°40’39’'N; verbatimLongitude: 105°57’22’’E; **Event:** samplingProtocol: trapping; eventDate: 6 Sept 2011; habitat: Karst forest; **Record Level:** collectionID: PNKB-NXN21; institutionCode: IEBR; collectionCode: DVZ-Rodentia
- **Type status:** Other material. **Occurrence:** recordedBy: Nghia Xuan Nguyen; individualCount: 1; sex: unkown; **Location:** country: Vietnam; stateProvince: Quang Binh; verbatimLocality: Thuong Hoa Commune, Minh Hoa District; verbatimElevation: 200-350m; verbatimLatitude: 17°40’05’'N; verbatimLongitude: 105°56’27’’E; **Event:** samplingProtocol: trapping; eventDate: 3 Sept 2011; habitat: Karst forest; **Record Level:** collectionID: PNKB-NXN232; institutionCode: IEBR; collectionCode: DVZ-Rodentia
- **Type status:** Other material. **Occurrence:** recordedBy: Nghia Xuan Nguyen; individualCount: 1; sex: unkown; **Location:** country: Vietnam; stateProvince: Quang Binh; verbatimLocality: Thuong Hoa Commune, Minh Hoa District; verbatimElevation: 200-350m; verbatimLatitude: 17°40’55’'N; verbatimLongitude: 105°54’40’’E; **Event:** samplingProtocol: trapping; eventDate: 7 April 2012; habitat: Karst forest; **Record Level:** collectionID: PNKB-NXN231; institutionCode: IEBR; collectionCode: DVZ-Rodentia
- **Type status:** Other material. **Occurrence:** recordedBy: Nghia Xuan Nguyen; individualCount: 1; sex: unkown; **Location:** country: Vietnam; stateProvince: Quang Binh; verbatimLocality: Thuong Hoa Commune, Minh Hoa District; verbatimElevation: 200-350m; verbatimLatitude: 17°40’32’'N; verbatimLongitude: 105°57’52’’E; **Event:** samplingProtocol: trapping; eventDate: 22 December 2013; habitat: Karst forest; **Record Level:** collectionID: PNKB-NXN181; institutionCode: IEBR; collectionCode: DVZ-Rodentia
- **Type status:** Other material. **Occurrence:** recordedBy: Nghia Xuan Nguyen; individualCount: 1; sex: Male; **Location:** country: Vietnam; stateProvince: Quang Binh; verbatimLocality: Thuong Hoa Commune, Minh Hoa District; verbatimElevation: 200-350m; verbatimLatitude: 17°40’07’'N; verbatimLongitude: 105°56’06’’E; **Event:** samplingProtocol: trapping; eventDate: 12 April 2014; habitat: Karst forest; **Record Level:** collectionID: PNKB-NXN219; institutionCode: IEBR; collectionCode: DVZ-Rodentia
- **Type status:** Other material. **Occurrence:** recordedBy: Nghia Xuan Nguyen; individualCount: 1; sex: Female; **Location:** country: Vietnam; stateProvince: Quang Binh; verbatimLocality: Thuong Hoa Commune, Minh Hoa District; verbatimElevation: 200-350m; verbatimLatitude: 17°40’27’'N; verbatimLongitude: 105°55’05’’E; **Event:** samplingProtocol: trapping; eventDate: 15 April 2014; habitat: Karst forest; **Record Level:** collectionID: Released to the wild
- **Type status:** Other material. **Occurrence:** recordedBy: Nghia Xuan Nguyen; individualCount: 1; sex: Female; **Location:** country: Vietnam; stateProvince: Quang Binh; verbatimLocality: Thuong Hoa Commune, Minh Hoa District; verbatimElevation: 200-350m; verbatimLatitude: 17°40’03’'N; verbatimLongitude: 105°56’27’’E; **Event:** samplingProtocol: trapping; eventDate: 15 April 2014; habitat: Karst forest; **Record Level:** collectionID: PNKB-NXN223; institutionCode: IEBR; collectionCode: DVZ-Rodentia
- **Type status:** Other material. **Occurrence:** recordedBy: Nghia Xuan Nguyen; individualCount: 1; sex: Female; **Location:** country: Vietnam; stateProvince: Quang Binh; verbatimLocality: Thuong Hoa Commune, Minh Hoa District; verbatimElevation: 200-350m; verbatimLatitude: 17°40’03’'N; verbatimLongitude: 105°56’27’’E; **Event:** samplingProtocol: trapping; eventDate: 18 April 2014; habitat: Karst forest; **Record Level:** collectionID: PNKB-NXN224; institutionCode: IEBR; collectionCode: DVZ-Rodentia
- **Type status:** Other material. **Occurrence:** recordedBy: Nghia Xuan Nguyen; individualCount: 1; sex: Female; **Location:** country: Vietnam; stateProvince: Quang Binh; verbatimLocality: Thuong Hoa Commune, Minh Hoa District; verbatimElevation: 200-350m; verbatimLatitude: 17°40’39’'N; verbatimLongitude: 105°57’34’’E; **Event:** samplingProtocol: trapping; eventDate: 5 May 2014; habitat: Karst forest; **Record Level:** collectionID: PNKB-NXN225; institutionCode: IEBR; collectionCode: DVZ-Rodentia
- **Type status:** Other material. **Occurrence:** recordedBy: Nghia Xuan Nguyen; individualCount: 1; sex: Male; **Location:** country: Vietnam; stateProvince: Quang Binh; verbatimLocality: Thuong Hoa Commune, Minh Hoa District; verbatimElevation: 200-350m; verbatimLatitude: 17°41’13’'N; verbatimLongitude: 105°53’51’’E; **Event:** samplingProtocol: trapping; eventDate: 27 June 2014; habitat: Karst forest; **Record Level:** collectionID: PNKB-NXN230; institutionCode: IEBR; collectionCode: DVZ-Rodentia

#### Description

Fig. 1

Fig. 2

#### Distribution

**Figure 3.**
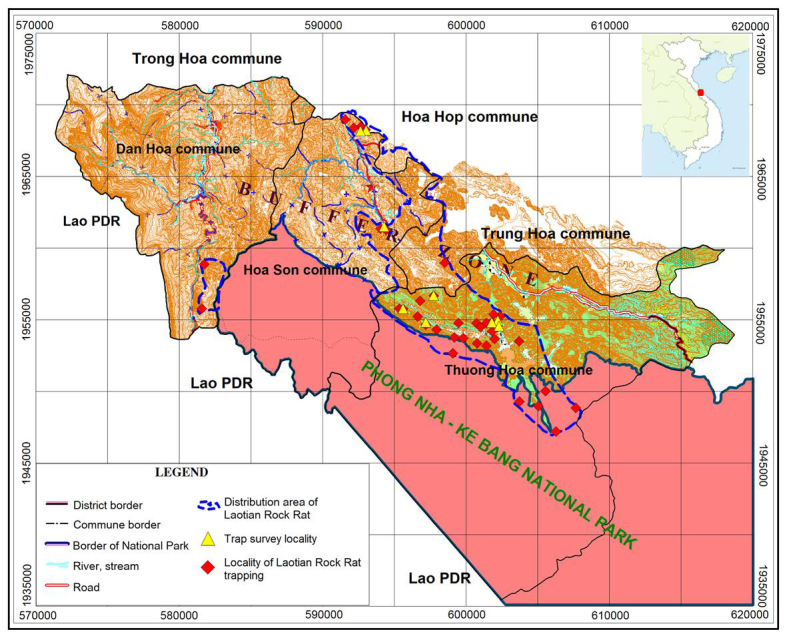
Distribution range of Laotian Rock Rat in PNKB NP, Quang Binh Province, Vietnam.

Interviews of local villagers indicated that the Laotian Rock Rat has been found at 35 localities in Thuong Hoa commune (24 localities), Hoa Son commune (9 localities) and Dan Hoa commune (2 localities) (Fig. 3). The interviewees also reported the occurrence of the Laotian Rock Rat in limestone forest extending from Thuong Hoa and Hoa Son communes to two neighboring communes, Trung Hoa and Hoa Hop. Cage traps and box traps were set up in 12 localities within these communes for a total of 9,050 trap-nights in April and May 2014. Only one live specimen of the Laotian Rock Rat was caught in Thuong Hoa commune (17°40'28”N; 105°55'05"E). This low trapping success can be explained by very low density of the Laotian Rock Rat in the survey area and because of dry season weather. As reported by local villagers, small mammal trapping success is always very low in dry season (from January to June). The live animal was released into the wild after taking its body measurements and monitoring it for a few days in semi-wild conditions. In total, during the period from the first reports of the Laotian Rock Rat in PNKB NP in September 2011 to October 2014, we collected 12 specimens of the species (one live animal and 11 dead specimens collected by local villagers) and checked coordinates of all these localities for the species distribution mapping. The distribution map of the Laotian Rock Rat was established based on data from the village interview surveys comprising 35 localities reported by villagers and 12 localities confirmed by our field surveys. The map showed that the distributional range of the Laotian Rock Rat in PNKB NP area covered about 150 km^2^ of limestone evergreen forests belonging to five communes (Thuong Hoa, Hoa Son, Trung Hoa, Hoa Hop and Dan Hoa) of Minh Hoa District, Quang Binh Province (Fig. 3).

#### Ecology

**Figure 4.**
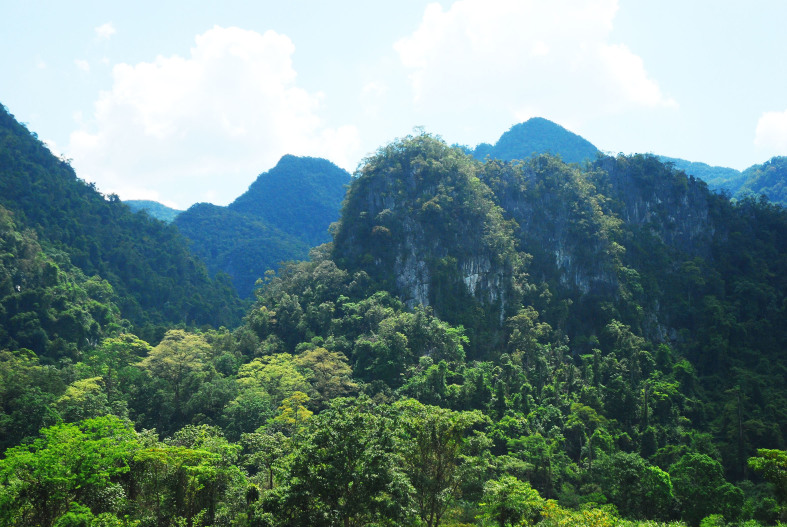
Moist limestone evergreen forest in LRR distribution area, PNKB NP, Vietnam.

Habitat and feeding ecology: The Laotian Rock Rat was found only in limestone evergreen forest on karst slopes (Fig. 4). This species has never been found in montane forest, forest in valleys, or in residential areas or in agricultural fields within the forests, but they can be found on karst slopes just 1-2 km away from villages or several hundred metres away from agricultural fields. The Laotian Rock Rat lives in the lower part of steep karst towers (100-300 m above valley base) with many rock boulders and crevices under tall limestone forest. The animals avoid living in the very low part of karst slopes where seasonal flooding occurs during the rainy season, and their dens are found far away from streams. Laotian Rock Rats use small rock crevices for their dens. During the day time, they hide in the dens, and at night they go out for foraging in forest floor next to the dens. The Laotian Rock Rat was not found in bare karst, but lives only in karst slopes under tall forest (moist evergreen forest) on limestone under 700 m asl (Fig. 4).

Most of the forested area has been affected by selected timber logging; however, a 3-4 layer forest structure remains, with the following characters:

The canopy tree layer consists of trees 20-25 m high with stem diameter 0.5-0.8 m. The most common trees species are: *Pometia pinnata* (Sapindaceae), *Dracontomelon duperreanum* (Anacardiaceae), *Toona surenii* (Meliaceae), *Paviesia anamensis* (Sapindaceae), *Pterospermum grewiaefolium* (Sterculiaceae), *Mahuca* sp., *Hopea* sp., *Streblus asper* (Moraceae), *Litsea* sp. (Lauraceae), *Sumbaviopsis macrophylla* (Euphorbiaceae), *Actinodaphne* sp. (Lauraceae), *Pometia chinensis* (Sapindaceae), *Choerospondias axillaris* (Anacardiaceae), *Alangium ridleyi* (Alangiaceae), *Knema* sp. (Myristicaceae); etc.

The middle tree layer consists of trees 10-15 m high with stem diameter of 0.3-0.5 m. The most common species are *Knema corticosa* (Myristicaceae); *Streblus tonkinensis*, *Streblus asper* (Moraceae), *Xylopia vielana* (Annonaceae), *Diospyros* sp. (Ebenaceae), *Caryota mitis* (Arecaceae), *Arenga pinnata* (Arecaceae), *Camelia* sp. (Theaceae), *Actinodaphne* sp. (Lauraceae), *Pterospermum* sp. (Sterculiaceae), *Litsea* sp.(Lauraceae), *Ormosia laoensis* (Fabaceae), *Nephelium lappaceum* (Sapindaceae), *Sumbaviopsis macrophylla* (Euphorbiaceae), *Paranephelium spirei* (Sapindaceae), *Alangium ridleyi* (Alangiaceae), *Baccaurea* sp. (Euphorbiaceae), *Aglaia* sp. (Meliaceae), etc.

The scrub layer consists of trees 3-7 m high, mostly with twisted stems, many branches, and several stems rising from one base. The most common species are from the families Euphorbiaceae, Theaceae, Myrtaceae and Verbenaceae. Some dominant species are *Antidesma* sp. (Euphorbiaceae), *Trevesia panmalta* (Araliaceae), *Litsea valiabilis* (Lauraceae), *Arenga pinnata* (Arecaceae), *Excoecaria cochinchinensis* (Euphorbiaceae), as well as seedlings of trees from higher layers.

The herb and fern layer is about 0.5-3 m high, with trees of 0.2-3 m high from family Araceae, the genera *Calamus* and *Caryota* (family Arecaceae), and many herb species from various families (Urticaceae, Melastomataceae, Balsaminaceae, Poaceae, Begoniaceae, Podipoliaceae, Convallariaceae, Zingiberaceae, Urticaceae, Acanthaceae). Some of the most common species are *Homalomena occulta* (Araceae), *Aglaonema simplex* (Araceae), *Aglaonema siamensis* (Araceae), *Tacca chantrieri* (Taccaceae), *Aspidistra typica* (Convallariaceae), *Piper* sp. (Piperaceae), *Corymborkis veratrifolia* (Orchidaeceae), etc.

Local villagers reported that the Laotian Rock Rat feeds exclusively on plant parts (leaves, roots and fruits) of various plant species. Stomach content analysis, examination of food remains dropped in dens, and observation of a live Laotian Rock Rat in semi-wild conditions showed that the species feeds mostly on plant parts, but also some insects, as follows:

- Young leaves and buds of *Aglaonema simplex* (Araceae), *Streblus asper* (Moraceae), *Dracontomelon duperreamum* (Anacardiaceae), *Pometia chinensis* (Sapindaceae), *Ficus* sp. (Moraceae), *Acanthopanax trifoliatus* (Araliaceae), *Perilla frutescens* (Lamiaceae), *Psidium guajava* (Myrtaceae), *Zea mays* (Poaceae), *Prunus persica* (Rosaceae)
- Ripe fruits of *Streblus asper* (Moraceae), *Ficus* sp. (Moraceae), *Dracontomelon duperreamum* (Anacardiaceae), *Alangium ridleyi* (Alangiaceae), *Lythocarpus fenestratus* (Fagaceae), *Musa paradisiaca*, *Musa uranoscapos* (Musaceae)
- Seeds of *Zea mays* (Poaceae)
- Roots of *Manihot esculenta* (Euphorbiaceae) and *Aglaonema simplex* (Araceae)
- Insects: Cicada (Cicadidae), Mantis (Mantidae), Grasshopper (Acrididae)

#### Conservation

The distribution range of the Laotian Rock Rat in PNKB NP is very small (approximately 150 km^2^) and located close to the villages of ethnic minorities (Ruc, Sach, Chut). These minority groups are very poor and their livelihood depends on wildlife and forest products. Wildlife hunting is a long tradition of the local people, and a practice that remains extensive currently. Most men 15 to 60 years in age in these villages are engaged in wildlife hunting. Their hunting season lasts about eight months per year (from July to February). The most widely used means for trapping the Laotian Rock Rat and other small animals is a metal spring snare, which has a high trapping success and can trap animals of various body size, such as rats, civets, large birds, and snakes. Snap traps are also used because they are easily made in the forest from bamboo and small trees. Each hunter usually keeps 30-100 active snares in forests; some hunters keep up to 300-500 active snares. It is estimated that 30-35 specimens of the Laotian Rock Rat are trapped by local villagers each year in the survey area. Other threats to the natural habitat of the Laotian Rock Rat include forest clearance for agricultural fields, removal of timber trees, collecting firewood and other forest products that lead to degradation of forest quality, and habitat modification. At present, no specific conservation measures are being undertaken aside from awareness education for local villagers.

## Discussion

**Distribution**

The confirmed distribution of the Laotian Rock Rat population in PNKB NP comprises roughly 150 km^2^ and is located in five communes of Minh Hoa District, Quang Binh Province, Central Vietnam. Topographically, the distribution range of the Laotian Rock Rat covers low karst mountains in these communes. The habitat quality of the limestone forest in the areas bordering to the east and to the north of this distributional area has been seriously disturbed and is not suitable for the Laotian Rock Rat. On the other hand, the area to the west of the species distribution range contains a vast area of limestone karst forest which continuously extends to Hin Namno National Biodiversity Area (NBCA) of Lao. It is expected that this area harbors the Laotian Rock Rat, but no surveys on rodents in this area were carried out.

In Lao, Vongsa (2010) indicated that the Laotian Rock Rat was found in five districts of Khammouane Province (Laos) within an area of 4,130 km^2^. The species was most abundant in Phou Hin Boun NBCA, while in two nearby limestone areas (Nam Kading NBCA to the north and Hin Namno NBCA to the east) there are no reports of the species. The distribution of the Laotian Rock Rat in PNKB NP, Vietnam is small and far away from Phou Hin Boun NBCA as well as the species distribution range in Khammuoane province. This implies that the population of the Laotian Rock Rat in PNKB NP may be genetically isolated from other populations in Lao and conservation measures for the Vietnamese population should be of high priority and urgency.

**Habitat**

Jenkins et al. (2005), Vongsa (2010), and Khotpathoom (2011) reported that in spite of several forest habitat types occurring in Phou Hin Poun NBCA (semi-evergreen forest, vine-bamboo forest, dry deciduous forest, mixed deciduous forest and wetlands), the Laotian Rock Rat was found only in limestone holes and in the vicinity of limestone caves at elevations from 263 to 734 m asl. The slopes surrounding karsts formations are covered with both evergreen trees and deciduous trees with little ground vegetation.

PNKB NP is located along the eastern slope of the Great Annamite Range which has a wetter climate. The forest habitat of PNKB NP is characterized by dense moist evergreen forest with different types such as evergreen forest on limestone above 700 m asl, montane evergreen forest on hills above 700 m asl, evergreen forest on limestone under 700 m asl, evergreen forest on hills under 700 m asl, degraded evergreen forest on hills, tree and scrub savanna on limestone, tree and scrub savanna on hills, agricultural land, and plantations and other land uses (Le et al. 2012). However, the Loatian Rock Rat was found only in evergreen forest on limestone karst under 400 m asl. These data indicate that the Laotian Rock Rat has a very narrow habitat preference which is restricted only to limestone karst covered by evergreen forest, semi-evergreen forest, and mixed deciduous forests.

**Threats and conservation**

Conservation of the Laotian Rock Rat is a high priority not only because the species is the only surviving member of the otherwise extinct family Diatomyidae that was formerly believed to have been extinct for more than 11 million years (Dawson et al. 2006) but also because both Lao's and Vietnam's populations have a very restricted distribution range and are facing high hunting pressure and habitat disturbance. The Laotian Rock Rat has been listed in the IUCN Red List as Endangered (Aplin and Lunde 2008) and in the Wildlife and Aquatic Red List of Lao (Khotpathoom 2011) for conservation action. In Vietnam, due to its recent discovery in 2011, the species has not been listed in the Red Data Book or any conservation legislative documents.

## Supplementary Material

XML Treatment for Laonastes aenigmamus

## Figures and Tables

**Figure 1.**
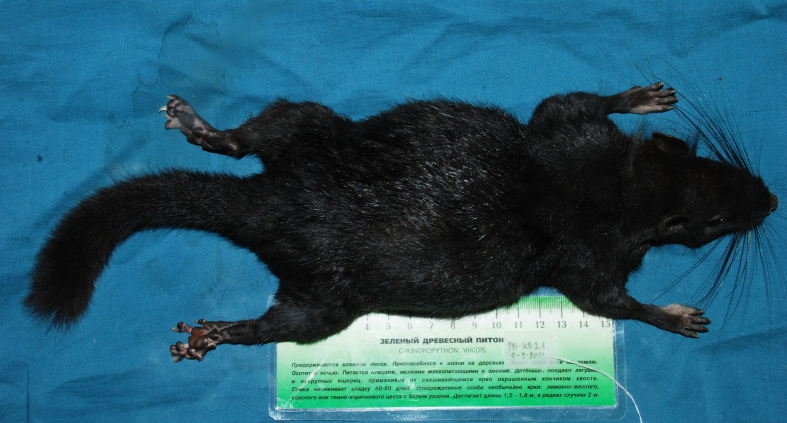
Dorsal view of the body of a Laotian Rock Rat *Laonastes aenigmamus*.

**Figure 2.**
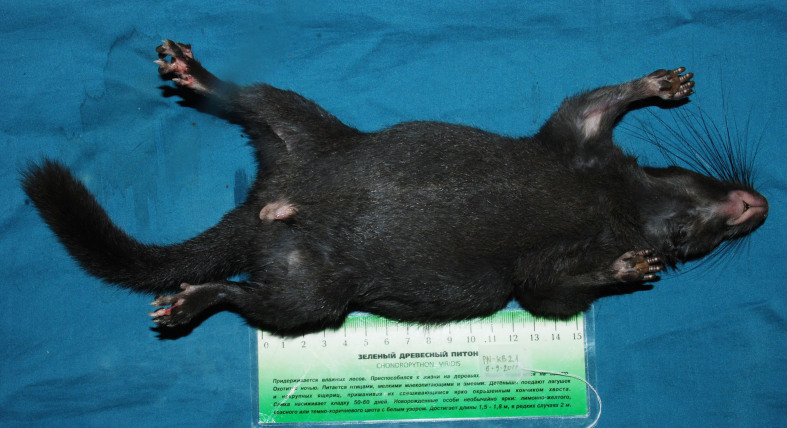
Ventral view of the body of a Laotian Rock Rat *Laonastes aenigmamus*.
